# The Diminishment of Novel Endometrial Carcinoma-Derived Stem-like Cells by Targeting Mitochondrial Bioenergetics and MYC

**DOI:** 10.3390/ijms23052426

**Published:** 2022-02-22

**Authors:** Laureen P. Helweg, Beatrice A. Windmöller, Leonie Burghardt, Jonathan Storm, Christine Förster, Nils Wethkamp, Ludwig Wilkens, Barbara Kaltschmidt, Constanze Banz-Jansen, Christian Kaltschmidt

**Affiliations:** 1 Department of Cell Biology, Faculty of Biology, University of Bielefeld, Universitätsstrasse 25, 33615 Bielefeld, Germany; beatrice.windmoeller@uni-bielefeld.de (B.A.W.); leonie.burghardt@uni-bielefeld.de (L.B.); jonathan.storm@uni-bielefeld.de (J.S.); barbara.kaltschmidt@uni-bielefeld.de (B.K.); c.kaltschmidt@uni-bielefeld.de (C.K.); 2 Forschungsverbund BioMedizin Bielefeld/OWL FBMB e.V., 33615 Bielefeld, Germany; christine.foerster@krh.eu (C.F.); ludwig.wilkens@krh.eu (L.W.); constanze.banz-jansen@evkb.de (C.B.-J.); 3 Institute of Pathology, KRH Hospital Nordstadt, Affiliated with the Protestant Hospital of Bethel Foundation, 30167 Hannover, Germany; nils.wethkamp@krh.eu; 4 Molecular Neurobiology, Faculty of Biology, Bielefeld University, 33615 Bielefeld, Germany; 5 Department of Gynecology and Obstetrics, and Perinatal Center, Protestant Hospital of Bethel Foundation, University Medical School OWL at Bielefeld, Bielefeld University, Campus Bielefeld-Bethel, 33615 Bielefeld, Germany

**Keywords:** cancer stem cells, endometrial cancer, primary endometrial cancer stem cells, MYC, metformin, mitochondria, EMT

## Abstract

Cancer stem cells (CSCs) are a small subpopulation of tumor cells harboring properties that include self-renewal, multi-lineage differentiation, tumor reconstitution, drug resistance and invasiveness, making them key players in tumor relapse. In the present paper, we develop new CSC models and analyze the molecular pathways involved in survival to identify targets for the establishment of novel therapies. Endometrial carcinoma-derived stem-like cells (ECSCs) were isolated from carcinogenic gynecological tissue and analyzed regarding their expression of prominent CSC markers. Further, they were treated with the MYC-signaling inhibitor KJ-Pyr-9, chemotherapeutic agent carboplatin and type II diabetes medication metformin. ECSC populations express common CSC markers, such as Prominin-1 and CD44 antigen as well as epithelial-to-mesenchymal transition markers, Twist, Snail and Slug, and exhibit the ability to form free-floating spheres. The inhibition of MYC signaling and treatment with carboplatin as well as metformin significantly reduced the cell survival of ECSC-like cells. Further, treatment with metformin significantly decreased the mitochondrial membrane potential of ECSC-like cells, while the extracellular lactate concentration was increased. The established ECSC-like populations represent promising in vitro models to further study the contribution of ECSCs to endometrial carcinogenesis. Targeting MYC signaling as well as mitochondrial bioenergetics has shown promising results in the diminishment of ECSCs, although molecular signaling pathways need further investigations.

## 1. Introduction

Endometrial carcinoma (EC) is the most common gynecological tumor and accounts for about 3% of worldwide mortality among women [1]. The main risk factors are obesity or diabetes mellitus, thus incidence rates are generally higher in high-income countries than in low- and middle-income countries [2]. Since 1983, EC has been broadly classified into two types, based on molecular profiling, histopathology and clinical behavior [3]; type I endometrial cancer accounting for 70–80% of all ECs is typically less aggressive, estrogen-related and highly-to-moderately differentiated with an endometrioid morphology. Type II endometrial cancer summarizes estrogen-independent non-endometrioid carcinomas [4]. ECs are usually diagnosed at an early stage due to symptoms that include postmenopausal uterine bleeding and have a good prognosis with a 5-year survival rate of 81.1% [5]. Recently, the results from The Cancer Genome Atlas (TCGA) project revealed four distinct molecular prognostic subgroups based on somatic copy number alterations (SCNAs) and tumor mutational burden that are now included in the molecular WHO Classification of Female Genital Tumors 2020: DNA polymerase-epsilon (POLE) ultramutated ECs, hypermutated ECs with microsatellite instability (MSI), ECs characterized by low copy numbers and mutational burden and ECs characterized by high copy numbers with frequent TP53 mutations [6,7]. The working group around López-Reig established a prognostic classification method using next generation sequencing of the most discriminant genes, which assigned the samples with an accuracy of nearly 100% [8].

With current cancer treatments, extensive and even complete cancer regression can be achieved, however this is often followed by relapse [9]. A key player driving tumor relapse is a small subpopulation of cancer cells that harbor properties that include self-renewal, multi-lineage differentiation, tumor reconstitution, drug resistance and invasiveness [10]. The presence of these so-called cancer stem cells (CSCs) has been assumed for several decades, as tumors recapitulate their corresponding tissue and hold a hierarchical organization with CSCs at the top. Endometrial carcinoma-derived stem-like cells (ECSCs) are commonly identified using specific cell surface markers, such as CD44-antigen (CD44) and Prominin-1 (CD133), as their expression is associated with tumorigenicity, invasiveness and metastasis [11,12,13]. Further, ECSCs were characterized by expressing stemness-related genes, such as MYC, SOX2, OCT4, ABCG2 and Nestin [14]. Accordingly, increased expression of ALDH1, OCT4, SOX2 and MYC was reported in a CD133^+^ cell subpopulation isolated from an endometrioid adenocarcinoma [15]. Consistently, downregulation of MYC in EC was shown to significantly reduce cell invasion as well as drug resistance [16]. Moreover, it has been suggested that CSCs arise from differentiated cancer cells through dedifferentiation mediated by epithelial-to-mesenchymal transition (EMT) [17]. EMT is a crucial process involved in embryonic development, which also contributes to cancer progression as cells gain stemness and chemoresistance as well as invasive characteristics (reviewed in [18]). Nuclear expression of the EMT transcription factor Snail as well as reduced E-cadherin in EC was found to be significantly associated with non-endometrioid histological type, FIGO stage, myometrial invasion, and patient survival, demonstrating that an EMT-positive status is related to poor overall survival and progression-free survival [19]. Furthermore, EMT controls metabolic reprogramming and the expression of different metabolic pathway genes, while metabolic dysregulation can induce EMT (reviewed in [20]). In recent years, several studies investigated the metabolic plasticity of CSCs as they are able to switch between increased oxidative mitochondrial metabolism and anaerobic glycolysis [21]. Accordingly, high mitochondrial mass correlates with aldehyde dehydrogenase (ALDH) activity, tumor-initiating activity and chemoresistance of CSCs [22].

Within this study, we established three ECSC-like cell populations named BKZ-10, BKZ-11 and BKZ-12, expressing various stemness-related proteins and harboring the ability to form spherical cancer organoids. The inhibition of the protein–protein interaction of MYC/NMYC with MYC-associated factor X (MAX), as well as the application of metformin and carboplatin, significantly decreased the cell survival of ECSCs. Further, metformin treatment significantly reduced mitochondrial membrane potential, representing metformin as a promising drug targeting the mitochondrial metabolism of ECSCs.

## 2. Results

### 2.1. Parental Tumor Classifications

Within this study, tumor materials from three female donors at the ages of 72, 83 and 86, all suffering from an endometrial adenocarcinoma, were sampled for the isolation of cancer stem-like cells. The parental tumor of the BKZ-10 and BKZ-11 cell populations showed no aberrant p53 expression, microsatellite stability and a wild-type POLE status, therefore exhibited no specific molecular profile and an intermediate-to-excellent prognosis (Table 1). BKZ-11 cells were isolated from a tumor that showed no aberrant p53 expression and a wild-type POLE status, but low frequent microsatellite instability. Therefore, this tumor was classified as an MMR-deficient EC with an intermediate prognosis (Table 1).

### 2.2. Successful Isolation of Endometrial Carcinoma-Derived Stem-like Cells

**Figure 1:**
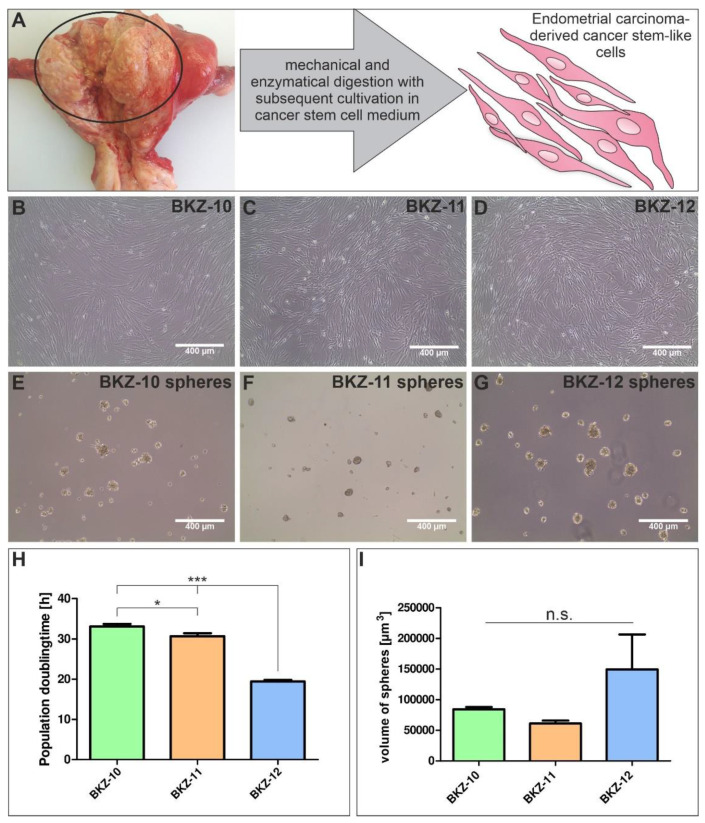
Establishment of endometrial carcinoma-derived stem-like cell populations. (**A**) For the isolation of endometrial carcinoma-derived stem-like cell populations, endometrial carcinoma tissue samples were obtained, mechanically and enzymatically digested and cultivated in cancer stem cell medium either supplemented with 10% fetal calf serum leading to (**B**–**D**) adherently growing cells or 4 µg/mL heparin for (**E**–**G**) sphere formation. (**H**) Quantification of population doubling time of BKZ-10, BKZ-11 and BKZ-12 revealed a significantly lower population doubling time for BKZ-12 in comparison to BKZ-10 and BKZ-11. Moreover, BKZ-10 exhibited a significantly higher population doubling time than BKZ-11. Quantification of the (**I**) volume of spheres showed a higher sphere volume for BKZ-12 in comparison to BKZ-10 and BKZ-11. Unpaired *t*-test (*p* ≤ 0.05). *n* = 3, * *p* ≤ 0.05, *** *p* ≤ 0.001. Means ± SEM (standard error of the mean).

To isolate cancer stem-like cells from carcinogenic endometrial tissues, the tumor samples were mechanically and enzymatically digested followed by cultivation in chemically defined CSC medium with added epidermal growth factor (EGF) and basic fibroblast growth factor (bFGF) (Figure 1A). Using serial trypsin treatment, we successfully cultivated adherently growing cells (Figure 1B–D) with the addition of fetal calf serum (FCS) as well as free floating spheres (Figure 1E–G) in serum free media for all three donors. Isolated ECSC populations depict an elongated spindle form morphology when cultured on 2D surfaces (Figure 1B–D).

The calculation of population doubling times revealed significant differences between each cell population. BKZ-10 depicted the highest population doubling time with 33.10 h (±0.65), which was slightly but significantly higher than the doubling time of BKZ-11 with a doubling time of 30.72 h (±0.76). BKZ12 proliferated significantly faster than the other two cell populations with an average population doubling time of 18.69 h (±0.34) (Figure 1H). Further, all cell populations were able to form organoid-like structures in the form of free-floating spheres with average sizes of 84,255, 61,164 and 149,576 µm^3^ for BKZ-10, BKZ-11 and BKZ-12, respectively (Figure 1I).

**Figure 2:**
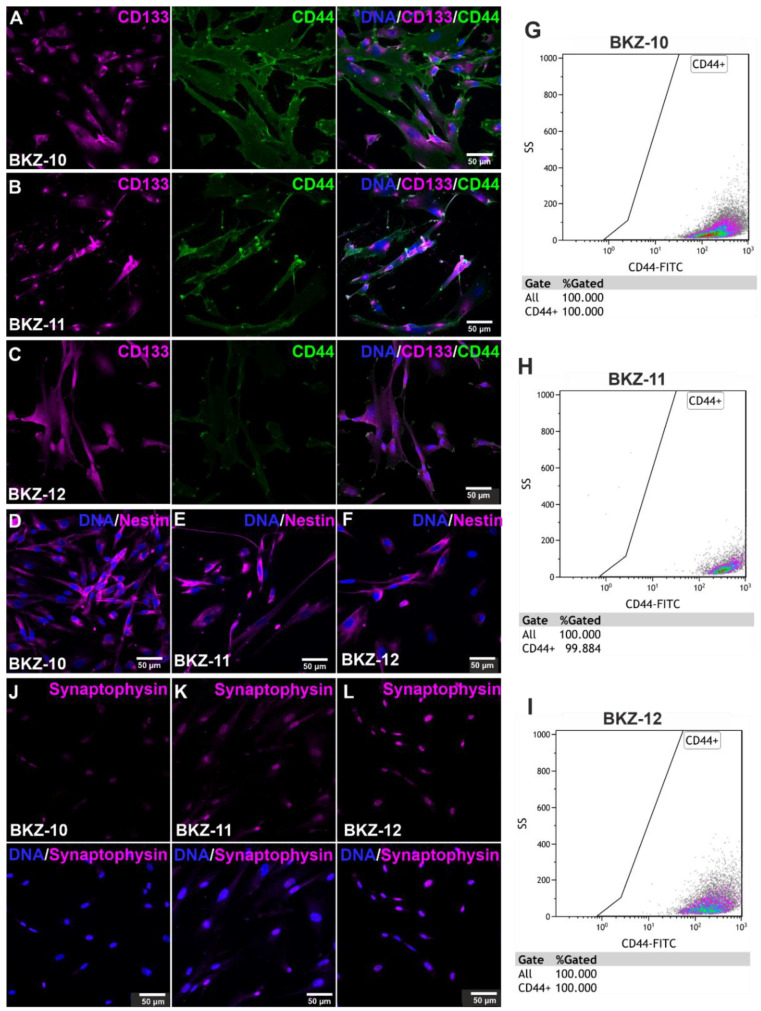
Isolated endometrial carcinoma-derived stem-like cell populations express cancer stem cell markers. Immunocytochemical staining revealed a high amount of cancer stem cell markers (**A**–**C**) Prominin-1 (CD133), CD44 antigen (CD44) and (**D**–**F**) Nestin. Flow cytometric analysis of CD44-positive cells depicted 100% positive cells for (**G**) BKZ-10 as well as for (**I**) BKZ-12 and 99.884% CD44-positive cells for (**H**) BKZ-11. Immunocytochemical analysis demonstrated the nuclear expression of synaptophysin for (**J**) BKZ-10, (**K**) BKZ-11 as well as (**L**) BKZ-12.

To confirm the isolation of ECSC populations, the protein expression of established CSC markers, CD44, CD133 and Nestin, was analyzed using immunocytochemistry and detected the robust expression of each marker for all three ECSC populations (Figure 2A–F).

Additional analysis via flow cytometry reveals 100% CD44-positive cells for BKZ-10 and BKZ-12 (Figure 2G,I) as well as 99.88% CD44-positive cells for BKZ-11 (Figure 2H). Fluorescence Minus One controls can be found in the supplement (Figure S1A–C). Further investigations regarding the neuroendocrine and novel CSC marker synaptophysin on protein level reveals a moderate expression of predominantly nuclear synaptophysin for BKZ-10, BKZ-11 and BKZ-12 (Figure 2J–L).

### 2.3. Endometrial Carcinoma-Derived Stem-like Cells Show an EMT Phentotype

**Figure 3:**
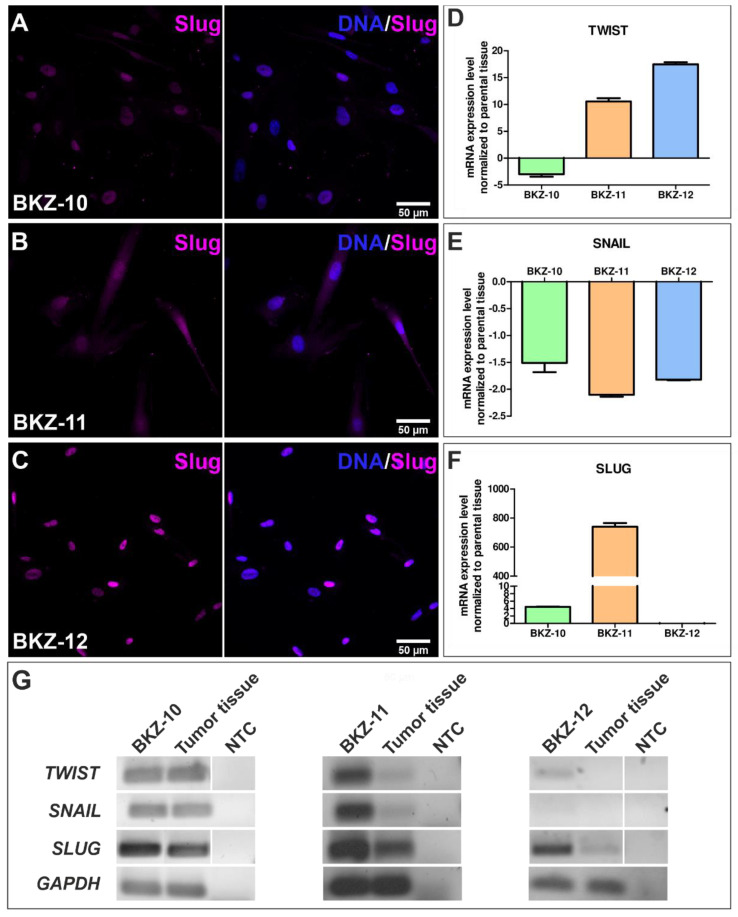
Endometrial carcinoma-derived stem-like cells express the epithelial-to-mesenchymal markers Twist, Snail and Slug. Immunocytochemical staining of Slug revealed a moderate nuclear protein expression in (**A**) BKZ-10 and (**B**) BKZ-11 as well as a high nuclear expression in (**C**) BKZ-12. Quantitative real-time PCR analysis of mRNA expression level normalized to the respective parental tissue mRNA expression level showed (**D**) enhanced TWIST expression for BKZ-11 as well as BKZ-12 and reduced expression for BKZ-10, (**E**) reduced SNAIL expression for all ECSC-populations and (**F**) enhanced SLUG expression for BKZ-10 and BKZ-11. Reverse transcriptase PCR (**G**) showed the mRNA expression of TWIST, SNAIL and SLUG for BKZ-10 and BKZ-11 and their respective parental tumor tissue, as well as TWIST and SLUG expression for BKZ-12 and SLUG expression for the parental tumor tissue of BKZ-12.

Next, we analyzed the expression of the EMT-related transcription factor Slug via immunocytochemistry and detected a moderate and high nuclear expression for BKZ-10 and BKZ-12, respectively (Figure 3A,C). For BKZ-11, a predominantly cytosolic expression of Slug was detected (Figure 3B).

The analysis of the EMT transcription factors Twist, Snail and Slug on transcriptional level using quantitative real-time PCR reveals a higher TWIST expression for BKZ-11 and BKZ-12, as well as a higher expression of SLUG for BKZ-10 and BKZ-11 normalized to the respective parental tissue mRNA expression (Figure 3D,F). However, the expression of SNAIL is lower in all ECSC populations (Figure 3E). The analysis of mRNA expression using reverse transcriptase PCR shows the expression of TWIST, SNAIL and SLUG for BKZ-10 and BKZ-12 as well as their respective tumor tissues (Figure 3G). BKZ-12 expresses TWIST and SLUG, while its parental tumor tissue solely expresses SLUG (Figure 3G).

**Figure 4:**
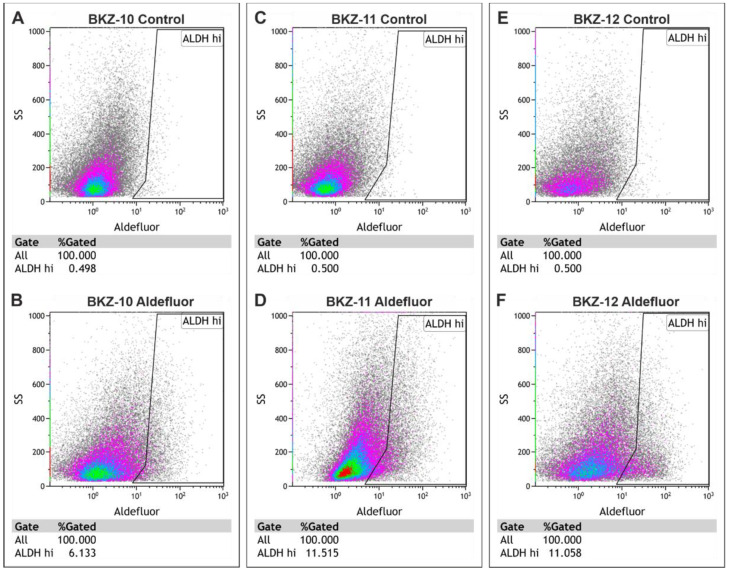
Established endometrial carcinoma-derived stem-like cell populations exhibit aldehyde dehydrogenase (ALDH) activity. Flow cytometric analysis of ALDH1 activity revealed (**B**) 6.133% ALDH high cells for BKZ-10, (**D**) 11.515% ALDH high cells for BKZ-11 and (**F**) 11.058% ALDH high cells for BKZ-12 in comparison to the respective control (**A**,**C**,**E**) with the specific ALDH inhibitor diethylaminobenzaldehyde (DEAB).

Further analysis of the ALDH activity of ECSCs reveals 6.13% ALDH high-expressing BKZ-10 cells, 11.51% ALDH high-expressing BKZ-11 cells and 11.06% ALDH high-expressing BKZ-12 cells (Figure 4).

### 2.4. Endometrial Carcinoma-Derived Stem-like Cells Reveal Immune-Evasive Characteristics

**Figure 5:**
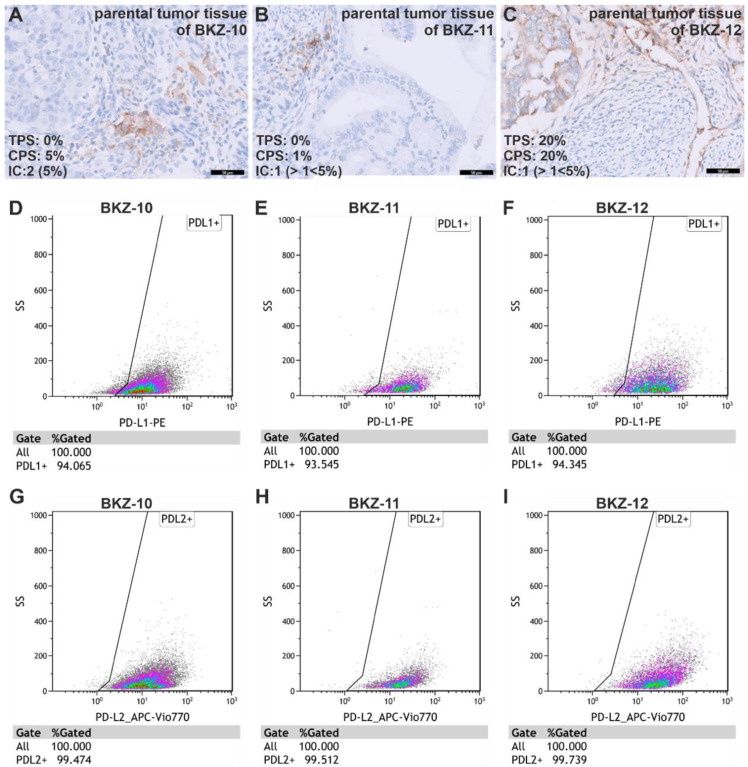
Isolated endometrial carcinoma-derived stem-like cell populations are enriched in PD-L1 and PD-L2 expression. (**A**) Immunohistochemical analysis of PD-L1 expression in the parental tissue of BKZ-10 revealed a tumor proportion score (TPS) of 0%, a combined positive score of 5% and an immune cell score (IC) of 2. (**B**) Analysis of PD-L1 expression in the parental tissue of BKZ-11 displayed a TPS of 0%, a CPS of 1% and an IC of 1. (**C**) For the parental tissue of BKZ-12, a TPS of 20%, a CPS of 20% and an IC of 1 was assessed. Analysis of PD-L1 (**D**–**F**) and PD-L2 (**G**–**I**) expression of endometrial carcinoma-derived cancer stem-like cell populations using flow cytometry revealed over 93% PD-L1-positive and 99% PD-L2-positive cells.

Histopathological examination of a bioptic sample retrieved from each parental tumor of the ECSC populations revealed immune checkpoint ligand programed death ligand 1 (PD-L1)-expressing cells in each sample. The analysis of PD-L1 expression was conducted according to international standards by calculating the combined positive score (CPS), tumor proportion score (TPS) and immune cell score (IC) (Formulas (4)–(6)). Quantification revealed 0% PD-L1-positive vital tumor cells for the parental tissue of BKZ-10, but analyzing the percentage of PD-L1-positive lymphocytes, macrophages and non-necrotic tumor cells by calculating the CPS displayed a score of 5%. Quantifying PD-L1-positive lymphocytes, macrophages, dendritic cells and granulocytes per tumor area depicts an IC score of 2 (Figure 5A). Similar to the parental tissue of BKZ-10, the analysis of PD-L1 expression of the parental tissue of BKZ-11 reveals no PD-L1-positive vital tumor cells. However, the CPS score for this tissue is 1% and the IC score is 1 (Figure 5B). Contrary to the first two ECs, the parental tissue of BKZ-12 depicts 20% PD-L1-positive vital tumor cells and a CPS score of 20% with an IC of 1 (Figure 5C).

Analysis of the expression of both PD-L1 and PD-L2 in ECSCs using flow cytometry reveals 94.06% PD-L1-positive BKZ-10, 93.55% PD-L1-positive BKZ-11 and 93.35% PD-L1-positive BKZ-12 cells (Figure 5D–F). Likewise, every ECSC-like cell population contains over 99% PD-L2-positive cells (Figure 5G–I). Fluorescence Minus One controls can be found in the supplement (Figure S1D–I).

### 2.5. Impairment of Endometrial Carcinoma-Derived Stem-like Cells by Targeting MYC

**Figure 6:**
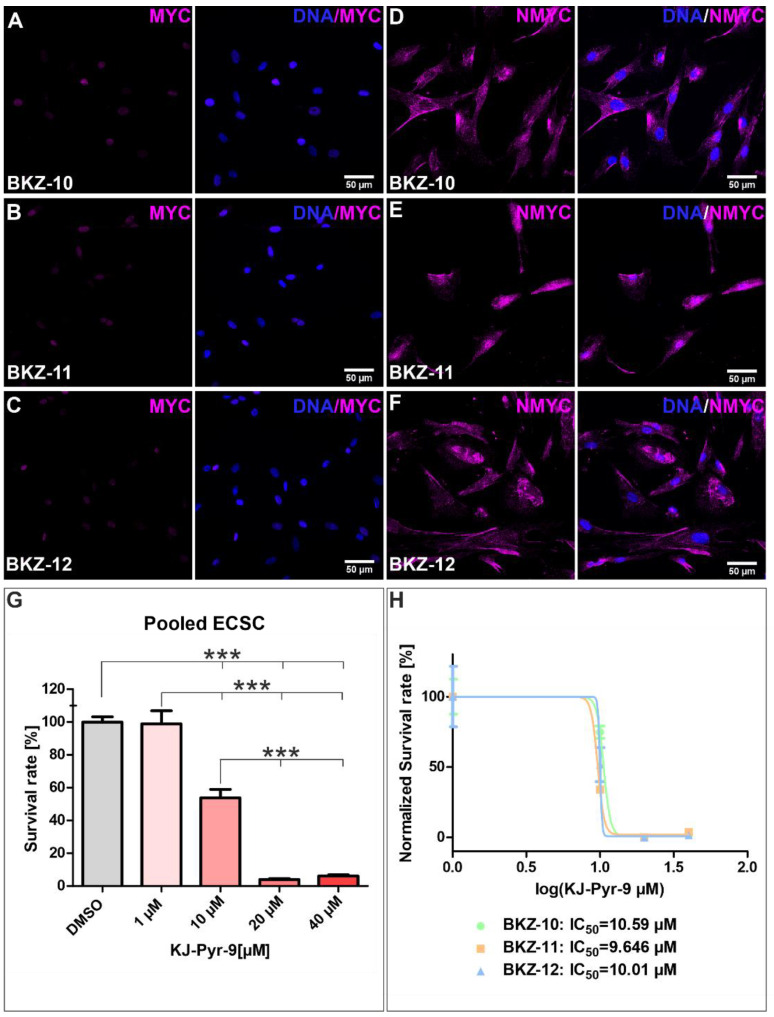
Inhibition of the MYC and NMYC protein interaction with MYC-associated factor X (MAX) decreases the survival of endometrial carcinoma-derived stem-like cell populations. Immunocytochemical staining revealed (**A**–**C**) predominantly nuclear MYC protein as well as (**D**–**F**) cytosolic NMYC protein for all three ECSC populations, respectively. To analyze the influence of the MYC/NMYC inhibitor KJ-Pyr-9, cells were treated with different concentrations for 120 h. Subsequent, viability was measured using Orangu^TM^ (Cell Guidance Systems, Cambridge, U.K., Cambridge, U.K.) and the cell count was determined by using a standard curve. (**G**) Statistical analysis revealed a significantly impaired survival of ECSCs upon application of 20, 40 and 60 µM KJ-Pyr-9. (**H**) Analysis of the half maximal inhibitory concentration (IC_50_) revealed an IC_50_ value of 10.59 µM KJ-Pyr-9 for BKZ-10, 9.646 µM KJ-Pyr-9 for BKZ-11 and 10.01 µM KJ-Pyr-9 for BKZ-12. Means ± SEM (standard error of the mean) were statistically analyzed by a non-parametric Mann–Whitney test (*n* = 3, *** *p* ≤ 0.001).

The proto-oncogenes MYC and NMYC are tightly regulated transcription factors and master regulators of various cellular processes. The analysis of MYC expression on protein level reveals the nuclear expression of MYC for each cell population (Figure 6A–C). Similarly, the robust expression of NMYC was detected for each cell population, even though the expression was predominantly cytosolic (Figure 6D–F).

To assess the influence of MYC and NMYC, cells were treated with an inhibitor of the protein–protein interaction of MYC/NMYC with MAX. Therefore, the small molecule KJ-Pyr-9 was applied and resulted in significantly decreased cell survival upon concentrations higher than 10 µM (Figure 6G). Further, the calculation of half maximal inhibitory concentration (IC_50_) values for each cell populations revealed similar results, with an IC_50_ value of 10.59 µM for BKZ-10, 9.646 µM for BKZ-11 and 10.01 µM for BKZ-12 (Figure 6H).

### 2.6. Metformin Targets the Mitochondrial Metabolism of Endometrial Carcinoma-Derived Stem-like Cells

**Figure 7:**
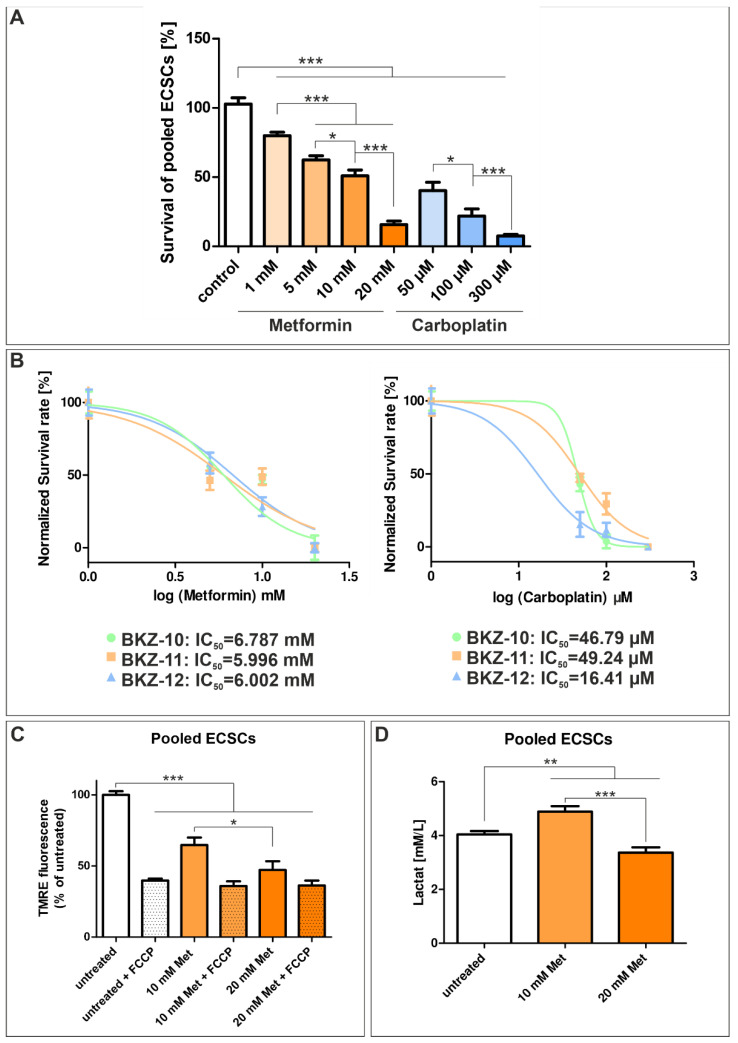
Treatment with metformin and carboplatin significantly decreases the survival of endometrial carcinoma-derived stem-like cell populations. To analyze the influence of carboplatin and metformin, the cells were treated with different concentrations of the reagents for 72 h. Afterwards, cellular viability was measured using Orangu^TM^ (Cell Guidance Systems, Cambridge, U.K., Cambridge, U.K.) and the cell count was determined by using the respective standard curve. (**A**) Statistical analysis of the survival rates of the pooled ECSCs revealed a significantly decreased survival of ECSCs for every tested concentration of carboplatin and metformin in a dose-dependent manner. (**B**) Analysis of the half maximal inhibitory concentration (IC_50_) for carboplatin revealed an IC_50_ value of 46.79 µM for BKZ-10, 49.24 µM for BKZ-11 and 16.41 µM for BKZ-12. The IC_50_ value for metformin was assessed as 6.787 mM for BKZ-10, 5.996 mM for BKZ-11 and 6.002 mM for BKZ-12. (**C**) Membrane potential of ECSCs was measured using TMRE, and the mitochondrial oxidative phosphorylation uncoupler FCCP was used as a technical control. Treatment with 10 and 20 mM metformin significantly reduces the membrane potential of ECSCs in comparison to untreated cells. (**D**) The lactate concentration of the media of untreated and treated cells was determined using Accutrend^®^ Plus, which revealed a significantly enhanced lactate concentration after treatment with 10 mM metformin and a significantly decreased concentration after treatment with 20 mM metformin. Means ± SEM (standard error of the mean) were statistically analyzed by an unpaired *t*-test (*n* = 3, * *p* ≤ 0.05, ** *p* ≤ 0.01, *** *p* ≤ 0.001).

Next to the influence of MYC/NMYC inhibition, the effect of standard type 2 diabetes medication metformin and chemotherapeutic agent carboplatin was assessed in ECSC populations. Already the lowest tested concentration of each drug significantly reduces the cell survival of ECSC populations (Figure 7A). In general, both drugs affected the cell survival of ECSC populations in a dose-dependent manner. Cells treated with 1 mM metformin show a survival rate of 79.92% (±2.47), while the application of 5 and 10 mM lead to survival rates of 62.47% (±1.93) and 50.96% (±4.26), respectively, and treatment with 20 mM depicts the most elevated survival decreasing effect with a survival rate of 15.65% (±2.67) (Figure 7A). Treatment with 50 µM carboplatin reveals an ECSC survival rate of 40.32% (±5.96), 100 µM of 21.98% (±5.13) and 300 µM of 7.61% (±1.07) (Figure 7A).

The calculation of IC_50_ for metformin depicts similar IC_50_ values for each cell population, as BKZ-10 depicts an IC_50_ value of 6.79 mM, BKZ-11 an IC_50_ value of 5.99 mM and BKZ-12 an IC_50_ value of 6.00 mM (Figure 7B). The IC_50_ values for carboplatin are more heterogeneous, as highly proliferative BKZ-12 is more sensitive with an IC_50_ value of 16.41 µM than BKZ-10 and BKZ-11 with IC_50_ values of 46.79 µM and 49.24 µM, respectively (Figure 7B). Even though carboplatin revealed the most effective survival-decreasing impact concerning the cumulated ECSC populations, metformin-mediated impairment was more consistent considering the different ECSC populations.

As metformin is known to modulate mitochondrial bioenergetics, the mitochondrial membrane potential of metformin-treated and -untreated cells was assessed using the mitochondrial membrane potential detecting TMRE staining. Treatment with 10 and 20 mM metformin significantly reduces the mitochondrial membrane potential of ECSCs (Figure 7C). Further, the lactate concentration of the media of ECSCs was measured, which was significantly enhanced after treatment with 10 mM metformin and significantly decreased after the application of 20 mM metformin (Figure 7D).

## 3. Discussion

Within this study, endometrial cancer-derived stem-like cell populations were successfully isolated and initially characterized as promising in vitro models to study ECSC-like cells. Treatment with the MYC-signaling inhibitor KJ-Pyr-9, diabetes medication metformin and chemotherapeutic agent carboplatin significantly reduced the cell survival of ECSCs. However, MYC inhibition and the usage of metformin depicted more coherent survival decreasing effects, demonstrating these as promising therapeutics in EC.

CSCs are a small subpopulation of highly tumorigenic cells that are characterized by the expression of markers, such as CD133, CD44 [12] and Nestin (reviewed in [23]). CD133^+^ EC cells showed an aggressive proliferation potential, colony formation and migration ability as well as higher chemoresistance [15]. Further, CD133^+^ ECSCs depicted an upregulation of CD44 [13], which is associated with infiltrating patterns and proliferation [11]. In addition to CD44, highly tumorigenic CD133^+^ ECSCs were shown to express Nestin [14] and the knockdown of Nestin inhibited cell growth, invasive potential and colony formation of EC cell lines, while the overexpression enhanced their malignant phenotype [24]. Another recently studied CSC marker is the neuroedocrine marker synaptophysin, whose nuclear expression was observed in CSCs derived from lung and colorectal cancer, as we recently published [25,26]. The co-expression of CSC markers CD133 and CD44 as well as Nestin and nuclear synaptophsin presented in this paper, strongly indicates the ECSC-like character and their potential as novel in vitro models to study EC-derived stem cells. The ability of all tested ECSC populations to form spheres further affirms their CSC-like phenotype, as ECSCs capable of forming sphere-like structures have an increased self-renewal and chemoresistance capacity as well as tumor initiating abilities in xenograft studies [13]. A study around Mori and coworkers revealed that ALDH-induced glycolysis mediates stemness and chemoresistance of EC-derived spheres [27]. However, the here-isolated ECSC populations only exhibited ALDH^high^ expressing cells at ranges between 6 and 11%. Accordingly, it was shown that breast CSCs balance between CD24^−^/CD44^+^ mesenchymal-like and ALDH^high^ epithelial-like stem cell populations, enabling reversible EMT/MET transitions [28]. This concords with the predominant expression of CD44 and the nuclear expression of a key EMT transcription factor, namely Slug, presented in this paper. Further, ECSCs express EMT transcription factor TWIST, which was also described as a potential target for vaccination against CSCs [29]. This expression of TWIST and SLUG in ECSCs emphasizes the correlation of EMT, stemness and the expression of programmed death ligand (PD-L) 1, as EMT has been suggested to induce the PD-L1-mediated immune evasion of CSCs [30]. Many cancers exhibit the ability to overcome immunosuppression, in which the expression of PD-L1 and -L2 play an important role [31,32]. PD-L1 expression has been suggested to modulate stem-like characteristics [33], chemoresistance, cell growth, stemness, drug resistance, immune evasion [34] and metastasis [35]. Moreover, instead of being induced by EMT, PD-L1 was also shown to promote an EMT phenotype [36]. A study around Kong and coworkers demonstrated that CD44 expression activated and therefore positively correlated with PD-L1 expression in breast and lung cancer [37]. Further, CD44 activation of PD-L1 was shown to be associated with immune infiltration, as depletion of CD44 in lung adenocarcinoma cells was linked with B cell, CD4+ T cell, neutrophil and dendritic cell infiltration [38]. Accordingly, flow cytometric analysis of the isolated 100% CD44-positive ECSC populations revealed over 93% positive PD-L1 and 99% PD-L2 expressing cells, substantiating the link between stemness and immune evasion of ECSCs. Accordingly, the proto-oncogene MYC has been shown to contribute to immunosuppression by directly inducing PD-L1 expression, while the downregulation of MYC led to immune cell infiltration (reviewed in [39]).

The MYC family consisting of MYC, NMYC and LMYC is a master regulator of various important cellular processes, thus playing a crucial role in tumorigenesis of various cancer types (reviewed in [40]). Expression of MYC was already observed in ECSC-like cells as well as EC-derived spheres [14,15,27], which concords with the here detected robust expression of MYC and NMYC. Recently, we assessed the global transcriptomes of CSCs derived from various tissues, including the ECSC populations (there referred to as ECSC_a, ECSC_b and ECSC_c) established in the present study and the detected co-expression of MYC and CD44 for BKZ-10 and BKZ-11 [41]. The depletion of MYC in EC cells has been shown to reverse Sal-like protein 4 (SALL4)-induced EMT, invasion and drug resistance [16]. Accordingly, we detected an EMT phenotype in the here-presented MYC-expressing ECSC populations, too. Further, we recently published a significant survival-decreasing effect through MYC-signaling inhibition utilizing small molecule KJ-Pyr-9 [42] in primary human colon and lung CSCs [26,43]. Consistently, the inhibition of MYC signaling in the here-established ECSC populations by application of KJ-Pyr-9 in concentrations greater than 10 µM significantly reduced cell survival. KJ-Pyr-9 has been shown to interfere with the heterodimerization of MYC and MAX by directly interacting with MYC, therefore disrupting its transcriptional activity [42]. The calculation of the IC_50_ revealed values around 10 µM, which stands in line with the recently reported IC_50_ values [43]. However, molecular signaling regarding KJ-Pyr-9-induced survival decrease needs further investigation to understand the underlying mechanism.

Next to KJ-Pyr-9, the effect of chemotherapeutic agent carboplatin and standard type 2 diabetes medication metformin on the established ECSC populations was assessed in the present study. A promising effect of the clinically used carboplatin in combination with paclitaxel was already shown in the treatment of advanced and recurrent EC [44]. Here, we demonstrate a significant survival decreasing effect of solely carboplatin on ECSCs. However, the responses were relatively heterogeneous with IC_50_ values differing between 16.41 µM for the highly proliferative BKZ-12 and 49.24 µM for BKZ-11. In line with carboplatin, the application of metformin impaired ECSC survival significantly but coherently for the three populations. Metformin has been shown to radiosensitize cancer cells and predominantly eradicate CSCs by the downregulation of genes, such as CD44 and EPCAM [45] or by the inhibition of EMT [46]. Notably, the calculated IC_50_ values around 6 mM are in accordance with the reported IC_50_ values of 3.72 and 6.77 mM [47]. Treatment of the EC cells with metformin was shown to promote progesterone receptor expression [48], which is often decreased in EC cell lines and together with low estrogen receptor (ER)-α expression associated with higher tumorigenicity [49]. On the contrary, metformin significantly downregulated ER-α and upregulated ER-β expression [50], which stands in line with the metformin-mediated inhibition of estradiol-induced EC cell proliferation and EMT [51]. Additionally, metformin has been shown to suppress migration in EC cell lines expressing Twist and Snail [52]. Further, metformin has been shown to inhibit cancer cell growth and induce apoptosis by suppressing mitochondrial-dependent biosynthetic activity [53]. The analysis of global transcriptomes of the here-established ECSC populations revealed an upregulation of genes associated with mitochondrial activity (referred to as ECSC_a, ECSC_b and ECSC_c) [41], while the application of metformin significantly impaired the mitochondrial membrane potential, highlighting the importance of mitochondrial respiration for ECSCs. As CSCs are able to switch to anaerobic glycolysis upon inhibition of mitochondrial metabolism [21], the increase in extracellular lactate concentration after the application of 10 mM metformin of ECSCs suggests a flexible metabolism to some extent. Nevertheless, they initially seem to prefer energy production through mitochondrial respiration, in contrast to the Warburg effect described in cancer cells [54,55]. As metformin is used as a standard type 2 diabetes medication and metabolic diseases as well as obesity may increase the risk of cancer, metformin use was associated with improved recurrence free and overall survival [56]. Here, peroxisome proliferator-activated receptors may link obesity and diabetes with cancer, as they are involved in many pathways, including energy production, combustion and storage as well as inflammation, cell cycle arrest, apoptosis, and DNA damage response (reviewed in [57,58]). Nevertheless, the potential link and underlying mechanisms have to be investigated in future studies.

**Figure 8:**
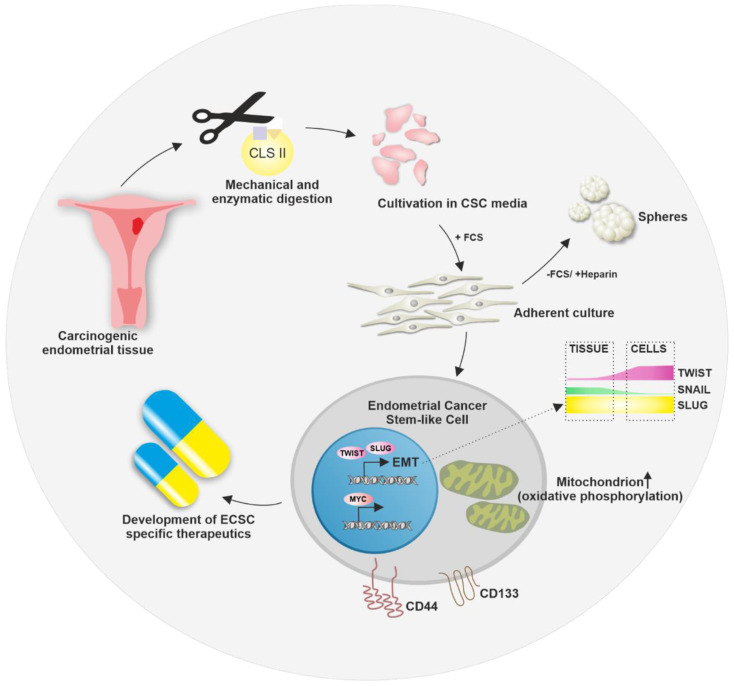
Establishment of endometrial cancer stem-like cells as new in vitro models. Carcinogenic endometrial tissue was sampled, mechanically and enzymatically digested and endometrial cancer stem-like cells were isolated by cultivation in CSC media. The adherent cells show CD133/CD44 co-expression, the expression of MYC and EMT key transcription factors TWIST and SLUG as well as mitochondrial metabolism. The novel endometrial cancer stem-like cells display a promising model for studying new cancer stem cell-specific therapeutics. CLS = collagenase; FCS = fetal calf serum; EMT = epithelial-to-mesenchymal transition.

In summary, we successfully established three novel primary ECSC-like cell populations as promising in vitro models to study CSC-mediated endometrial carcinogenesis. The CSC-like phenotype was confirmed by its expression of CD133, CD44, Nestin and synaptophysin as well as sphere formation (Figure 8). Additionally, ECSCs exhibited ALDH activity and expressed EMT markers as well as proto-oncogenes MYC and NMYC, further emphasizing stem-like properties. The inhibition of MYC signaling as well as the application of metformin and carboplatin led to a significant reduction of cell survival of ECSCs, while the usage of the MYC inhibitor KJ-Pyr-9 and metformin acted more uniformly on the different ECSC populations. The inhibition of MYC signaling at low doses of KJ-Pyr-9 led to similar survival rates as application of metformin and carboplatin at much higher concentrations. Further, metformin was shown to significantly decrease mitochondrial membrane potential and increase lactate concentration, indicating the metabolic plasticity of ECSCs. Thus, targeting MYC signaling and mitochondrial bioenergetics represent promising novel options for the treatment of EC affecting ECSCs.

## 4. Materials and Methods

### 4.1. DNA Extraction and MSI Analysis

Formalin-fixed and paraffin-embedded endometrium carcinoma and non-neoplastic tissues were manually microdissected under microscopic control. For tumor tissues, areas were used where a high-tumor cell concentration (at least 50% tumor cell content) had been microscopically identified by a certified pathologist. Genomic DNA was extracted using the Maxwell RSC machine with the Maxwell RSC DNA FFPE Kit according to the manufacturer’s instructions (Promega, Madison, WI, USA). MSI analysis was performed as previously described [59]. Briefly, the five microsatellite loci (BAT-25, BAT-26, D2S123, D5S346 and D17S250) recommended by the 1997 NCI-sponsored MSI workshop were amplified in two separate multiplex PCR reactions using fluorescent dye-labeled oligonucleotides. Then, PCR products were analyzed by capillary electrophoresis using an ABI 3500 Genetic Analyzer and GeneMapper 6 software (Thermo Fisher Scientific, Bremen, Germany). For interpretation purposes, microsatellite instability at ≥2 loci was defined as MSI high (MSI-H) and if no instability at any of the loci tested was defined the sample was considered as MSS (stable). In case instability at one of the loci tested was identified, the sample was further analyzed using a second MSI panel composed of the markers BAT-40, D18S58, D13S153, D10S197 and Myc-L. Again, amplification was carried out using two separate multiplex PCR reactions followed by capillary electrophoresis, as mentioned above. Then, the samples with instability in >3 out of 10 loci were defined as MSI-H while samples with instability in 1–3 out of 10 loci were classified as MSI low (MSI-L) [60].

### 4.2. POLE Mutation Analysis

Tumor DNA samples were analyzed for mutations in exons 9, 10, 11, 12, 13 and exon 14 of POLE (NG_033840.1) by Sanger sequencing. Exons were amplified using M13-tailed oligonucleotides, as previously published [61,62]. After the purification of PCR products using the QIAquick PCR Purification Kit (Qiagen, Hilden, Germany), bidirectional Sanger sequencing of PCR products was performed on an ABI 3500 Genetic Analyzer (Thermo Fisher Scientific, Bremen, Germany) using the BigDye Terminator v3.1 Cycle Sequencing Kit (Thermo Fisher Scientific, Bremen, Germany) according to standard protocols. Finally, sequence analysis was carried out using SeqScape 4 software (Thermo Fisher Scientific, Bremen, Germany).

### 4.3. Endometrial Cancer Stem-like Cell Establishment and Cell Culture

The cancer tissue samples used for the isolation of ECSCs were obtained during surgical resection after assuring routine histopathological analysis and were kindly provided by the Forschungsverbund BioMedizin Bielefeld/OWL FBMB e. V. (Bielefeld, Germany) at the Protestant Hospital of Bethel Foundation (Bielefeld, Germany). Informed consent according to local and international guidelines was signed by all patients and further experimental procedures were ethically approved (Ethics committee Münster, Münster, Germany, 2017-522-f-S).

For the isolation of primary cells, a cubic tumor material sample measuring 5 mm was collected from each tumor and transferred to ice-cold Dulbecco’s Phosphate Buffered Saline (PBS; Sigma-Aldrich, Munich, Germany), repeatedly washed with PBS, mechanically disintegrated and enzymatically digested with collagenase for 2 h at 37 °C, as previously described [26,41,43]. The minced tissue was cultivated in CSC medium containing Dulbecco’s Modified Eagle’s medium/Ham’s F-12 (Sigma-Aldrich, Munich, Germany), 2 mM l-glutamin (Sigma-Aldrich, Munich, Germany), penicillin/streptomycin (100 μg/mL; Sigma-Aldrich, Munich, Germany), EGF (20 ng/mL; Miltenyi Biotec, Bergisch Gladbach, Germany), bFGF (40 ng/mL; Miltenyi Biotec, Bergisch Gladbach, Germany), B27 supplement (Gibco, Thermo Fisher Scientific, Bremen, Germany), supplemented with 10% FCS (Sigma-Aldrich, Munich, Germany) on T75 culture flasks coated with 0.1% gelatin from bovine skin (type-B; Sigma-Aldrich, Munich, Germany). CSCs were enriched through serial trypsin treatment, as described by Walia and coworkers and Morata-Tarifa and colleagues [63,64]. Briefly, after washing with PBS, the cells were treated with a 0.05% trypsin-EDTA solution (0.5 mg/mL; Sigma-Aldrich, Munich, Germany) for 5 min and transferred onto a new gelatin-coated culture flask. To assure stem-like characteristics, trypsinization was repeated every 48 to 72 h for at least three cycles. For sphere formation, 0.5 × 10^6^ cells were cultured in serum-free CSC medium supplemented with 4 μg/mL heparin (Sigma-Aldrich, Munich, Germany) in low adhesion culture flasks. All of the cells were cultured at 37 °C and 5% CO_2_ in a humidified incubator.

Population doubling times were determined using the Orangu^TM^ Cell Counting Solution (Cell Guidance Systems, Cambridge, U.K.) according to the manufacturer’s guidelines. As a standard curve, 1000, 2500, 5000, 7500 and 10,000 cells and to determine the population doubling time 3000 cells per 100 μL CSC medium supplemented with 10% FCS were seeded in a 0.1% gelatin-coated 96 well-plate. After adherence, cell viability was measured for the standard curve and after 72 h for the growing cells. The cell count was quantified using the respective standard curve, growth rate and the population doubling times were determined by the following equations:

(1) $$\mathrm{growth}\;\mathrm{rate}=\frac{\ln\left(xt\right)-\ln\left(x0\right)}{t-t0}$$

(2) $$\mathrm{population}\;\mathrm{doubling}\;\mathrm{time}=\frac{\ln\left(2\right)}{\mathrm{growth}\;\mathrm{rate}\;}$$

To quantify the volume of the spheres, at least five pictures were taken and every sphere was measured in length and width using Fiji ImageJ [65]. The volume of the spheres was calculated according to the following equation:

(3) $$V=\frac{4}{3}\times \pi \times {\left(\frac{\mathrm{sphere}\;\mathrm{diameter}}{2}\right)}^{3}$$

### 4.4. Immunocytochemistry

For immunocytochemical staining, pre-cultured cells were harvested and seeded on etched cover slips in a 24-well plate at a density of 1.5 × 10^4^ cells per 500 µL CSC medium supplemented with 10% FCS. At 80% confluency, the cells were fixated with 4% phosphate-buffered paraformaldehyde (lab-made) for 15 min at room temperature (RT), washed with PBS, blocked/permeabilized using 0.02% Triton-X 100 (Sigma-Aldrich, Munich, Germany) with 5% goat serum (Dianova, Hamburg, Germany) for 30 min at RT and incubated with the primary antibody for 1 h at RT. The antibodies used were anti-CD44 (1:400; 156-3C11; Cell Signaling, Frankfurt am Main, Germany), anti-CD133 (1:100; NB120-16518; NovusBio, Bio-Techne, Wiesbaden-Nordenstadt, Germany), anti-Nestin (1:200; MAB5326; Sigma-Aldrich, Munich, Germany), anti-MYC (0.1 μg/mL; Y69; Abcam, Cambridge, U.K.), anti-NMYC (2.5 μg/mL; NCM II 100; Abcam, Cambridge, U.K.), anti-synaptophysin (1:250; MAB5258; Sigma-Aldrich, Munich, Germany) and anti-Slug (1:100; C19G7; Cell Signaling, Frankfurt am Main, Germany). Secondary fluorochrome-conjugated antibodies (1:300; goat anti-mouse Alexa 555, goat anti-rabbit Alexa 555, goat anti-mouse Alexa 488; Life Technologies, Thermo Fisher Scientific, Bremen, Germany) were incubated for 1 h at RT in the dark. Nuclear counterstaining was performed with 4′,6-diamidino-2-phenylindole (1 μg/mL; Sigma-Aldrich, Munich, Germany) for 10 min at RT. Fluorescence imaging was performed using a confocal laser scanning microscope (LSM 780; Carl Zeiss, Jena, Germany) and analyzed using ZEN software from the same provider or Fiji ImageJ [65].

Immunohistochemical stainings were performed at the Institute of Pathology of KRH Hospital Nordstadt (Hannover, Germany) using the automated immunohistochemistry and in situ hybridization platform DAKO Omnis (DAKO, Agilent, Santa Clara, CA, USA), according to the manufacturer’s instructions. The used antibodies were anti-PDL1 (22C3; DAKO, Agilent, Santa Clara, CA, USA) and anti-p53 (DO-7, DAKO, Agilent, Santa Clara, CA, USA). For the anti-p53 staining, slides were pre-treated in citrate (pH 6) for 30 min. For visualization, the EnVision FLEX, high pH (DAKO, Agilent, Santa Clara, CA, USA) visualization system was used according to the manufacturer’s instructions. The analysis of the expression of PDL1 was performed according to international standards [66]:

(4) $$\mathrm{TPS}=\frac{\mathrm{PDL}1\;\mathrm{positive}\;\mathrm{vital}\;\mathrm{tumor}\;\mathrm{cells}}{\mathrm{PDL}1\;\mathrm{positive}+\mathrm{negative}\;\mathrm{vital}\;\mathrm{tumor}\;\mathrm{cells}}$$

(5) $$\mathrm{CPS}=\frac{\mathrm{PDL}1\;\mathrm{positive}\;\mathrm{vital}\;\mathrm{tumor}\;\mathrm{cells}+\mathrm{lymphocytes}+\mathrm{macrophages}}{\mathrm{PDL}1\;\mathrm{positive}+\mathrm{negative}\;\mathrm{vital}\;\mathrm{tumor}\;\mathrm{cells}}\times 100$$

(6) $$\mathrm{IC}=\frac{\mathrm{PDL}1\;\mathrm{positive}\;\mathrm{lymphocytes}+\mathrm{macrophages}+\mathrm{dendritic}\;\mathrm{cells}+\mathrm{granulocytes}}{\mathrm{tumor}\;\mathrm{area}}$$

### 4.5. Flow Cytometry

Cultured cells were harvested and stained with anti-CD44 FITC (P-glycoprotein 1; 1:50; REA690; Miltenyi Biotec, Bergisch Gladbach, Germany), anti-CD273 APC-Vio^®^770 (PDCD1-L2; 1:50; REA985; Miltenyi Biotec, Bergisch Gladbach, Germany) and anti-CD274 PE (PDCD1-L1; 1:10; MIH1; BD Biosciences, Heidelberg, Germany). Dead cells were excluded via propidium iodide (PI; 1 µg/mL; Thermo Fisher Scientific, Bremen, Germany). Analysis was performed using a Gallios Flow Cytometer and the Kaluza 1.0 software (both Beckman Coulter Life Sciences, Krefeld, Germany) using appropriate Fluorescence Minus One controls and automatic compensation. Unspecific binding was controlled for using appropriate isotype controls beforehand. The measurements of ALDH activities were performed utilizing the ALDEFLUOR™ Kit (STEMCELL Technologies, Vancouver, BC, Canada) according to the manufacturer’s guidelines and PI (1 µg/mL; Thermo Fisher Scientific, Bremen, Germany) for dead-cell discrimination with the instrument and software mentioned above.

### 4.6. Polymerase Chain Reaction

For the analysis of mRNA expression, RNA was isolated using the NucleoSpin RNA Kit (Macherey Nagel, Düren, Germany) according to the manufacturer’s guidelines. cDNA synthesis was performed using 1 µg RNA and the First Strand cDNA Synthesis Kit (Thermo Fisher Scientific, Bremen, Germany). The quantification of RNA was performed using the Primers listed below (Table 2) and the Platinum SYBR Green qPCR Super-Mix UDG (Invitrogen, Thermo Fisher Scientific, Bremen, Germany) according to the manufacturer’s guidelines and assayed with a Rotor Gene 600 (Qiagen, Hilden, Germany). Reverse transcriptase PCR was performed using the *Taq* DNA Polymerase with ThermoPol^®^ Buffer (New England Biolabs, Frankfurt am Main, Germany) and the primers listed below according to the manufacturer’s guidelines. Amplified RT-PCR products were separated on an 2% agarose gel (Sigma-Aldrich, Munich, Germany) with 0.001% ethidium bromide (Carl Roth GmbH, Karlsruhe, Germany) run at 100 V, and were imaged with a trans-illuminator (UVsolo TS; Biometra, Göttingen, Germany).

### 4.7. Inhibitor and Drug Treatments

ECSCs were treated with the MYC/NMYC inhibitor KJ-Pyr-9 (Merck, Darmstadt, Germany), diabetes medication metformin (Merck, Darmstadt, Germany) and chemotherapeutic agent carboplatin (Sigma-Aldrich, Munich, Germany). Cell viability was assayed using Orangu^TM^ Cell Counting Solution according to the manufacturer’s instructions. For the standard curve, 1000, 2500, 5000, 7500 and 10,000 cells and for the treatment 3000 cells per 100 µL CSC medium supplemented with 10% FCS were seeded in a 0.1% gelatin-coated 96 well-plate. After adherence, cell viability was measured for the standard curve and the treatment started by applying KJ-Pyr-9 in the concentrations of 10 µM, 20 µM, 40 µM and 60 µM plus solvent control, metformin in the concentrations of 1, 5, 10 and 20 mM and carboplatin in the concentrations of 50, 100 and 300 µM. After treatment with metformin and carboplatin for 72 h and KJ-Pyr-9 for 96 h, cell viability was measured and cell count quantified using the respective standard curve. Relative survival rates were determined by normalizing each cell count to the mean of controls for the respective cell population and the half maximal inhibitory concentration (IC_50_) calculated from a log(concentration) versus normalized survival rate non-linear regression fit using Prism V5.01 software (GraphPad Software, Inc., San Diego, CA, USA).

### 4.8. Mitochondrial Membrane Potential Assay and Lactate Concentration

To analyze the mitochondrial membrane potential and assess the lactate concentration, 3000 cells per 100 µL CSC medium supplemented with 10% FCS were seeded in a 0.1% gelatin-coated 96 well-plate and after adherence 10 and 20 mM metformin applied. After 72 h, the mitochondrial membrane potential was assessed using the TMRE Mitochondrial Membrane Potential Kit (Abcam, Cambridge, U.K.) according to the manufacturer’s instructions. Briefly, 20 µM FCCP was added to the respective control wells 10 min prior to TMRE staining with 400 nM TMRE for 25 min. The medium was discarded, the cells were washed with 100 µL 0.2% BSA/PBS (Sigma-Aldrich, Munich, Germany), 100 µL 0.2% BSA/PBS was added to the cells and the fluorescence read at Ex525/Em580. To measure the lactate concentration of the media of treated and untreated ECSCs, 25 µL of the discarded medium was applied on the Accutrend^®^ Plus using the BM-Lactate test strips (Roche, Mannheim, Deutschland).

### 4.9. Statistical Analysis

Data were raised at least in triplicate and statistically analyzed using the Prism V5.01 software (GraphPad Software, Inc., San Diego, CA, USA). The test for normality was conducted using D’Agostino and Pearson omnibus normality test. To evaluate the differences between multiple groups, the Student’s *t*-test or non-parametric Mann–Whitney test were performed. A significance value of *p* ≤ 0.05 was considered as statistically significant. The data are presented as the means ± standard error of the mean (SEM).

## Figures and Tables

**Table 1:** Molecular classification of parental endometrial adenocarcinomas. IHC = immunohistochemistry; MSS = microsatellite stable; MSI-L = microsatellite instability—low.

Donor of	P53-IHC	Mismatch–Repair	POLE Status	Classification	Prognosis
**BKZ-10**	Not aberrant	MSS	Wild type	NSMP EC = no specific molecular profile	Intermediate to excellent
**BKZ-11**	Not aberrant	MSI-L	Wild type	MMR-deficient EC	Intermediate
**BKZ-12**	Not aberrant	MSS	Wild type	NSMP EC = no specific molecular profile	Intermediate to excellent

**Table 2:** Primers used for qPCR.

Target Gene	Sequence
Beta-Aktin (AKTB) forward	TCCCTGGAGAAGAGCTACGA
AKTB reverse	AGCACTGTGTTGGCGTACAG
Glyceraldehyde 3-phosphate dehydrogenase (GAPDH) forward	CATGAGAAGTATGACAACAGCCT
GAPDH reverse	AGTCCTTCCACGTATACCAAAGT
Ribosomal protein lateral stalk subunit P0 (RPLP0) forward	TGGGCAAGAACACCATGATG
RPLP0 reverse	AGTTTCTCCAGAGCTGGGTTGT
Snail (SNAI1) forward	CCCAATCGGAAGCCTAACTA
SNAI1 reverse	GGACAGAGTCCCAGATGAGC
Slug (SNAI2) forward	TCGGACCCACACATTACCTT
SNAI2 reverse	TTGGAGCAGTTTTTGCACTG
Twist-related protein 1 (TWIST1) forward	GTCCGCAGTCTTACGAGGAG
TWIST1 reverse	CCAGTTGAGGGTCTGAATC

## Data Availability

Not applicable.

## Supplementary Materials

The following supporting information can be downloaded at: https://www.mdpi.com/article/10.3390/ijms23052426/s1.
