# Supplementary figures and images for: The Diminishment of Novel Endometrial Carcinoma-Derived Stem-like Cells by Targeting Mitochondrial Bioenergetics and MYC
Source: Int J Mol Sci. 2022 Feb 22;23(5):2426. doi: 10.3390/ijms23052426 (PMC8910063; doi:10.3390/ijms23052426)

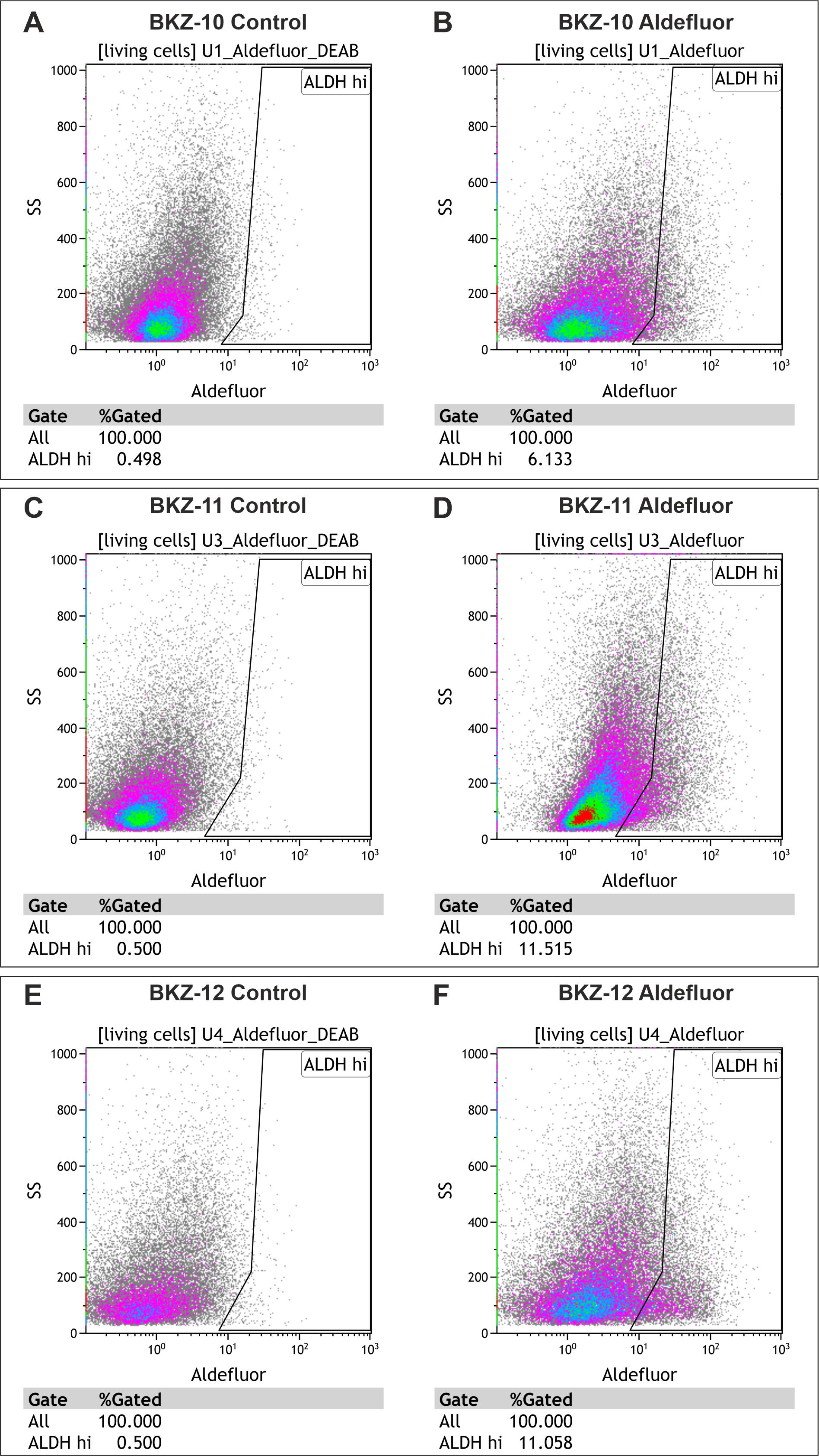

Supplement: Supplementary file 1 [file ijms-23-02426-s001.zip › Supplement/Figure S1.png]
